# Concurrent Oral Squamous Cell Carcinoma and Incidental Papillary Thyroid Carcinoma Metastasis in Cervical Lymph Nodes: A Case Report

**DOI:** 10.7759/cureus.66413

**Published:** 2024-08-07

**Authors:** Adhithya Baskaran, Shamala Ravikumar, Kokila Sivakumar, Janani Ilango, Radhika Chelliah

**Affiliations:** 1 Department of Oral Pathology, Adhiparasakthi Dental College and Hospital, Melmaruvathur, IND

**Keywords:** lymph node metastasis, metastatic thyroid carcinoma, head and neck squamous cell carcinoma, papillary thyroid carcinoma, follicular type papillary thyroid carcinoma (ptc), poorly differentiated squamous cell carcinoma

## Abstract

Oral squamous cell carcinoma (OSCC) is the most common of all head and neck cancers accounting for 90% of all oral malignancies. It is commonly associated with the use of tobacco smoking or quid form. The incidence of oral carcinoma is higher in males than females with a ratio of 1.4:1, though females commonly adopt tobacco quid chewing habit. OSCC metastasis to cervical lymph node at the rate of 20-42.6% according to studies reported so far. Papillary thyroid carcinoma (PTC) occurs with a higher incidence in females than males with metastasis into cervical lymph nodes though the primary lesion frequently goes undetected. Concurrent metastasis of OSCC and PTC to the cervical lymph node during neck dissection has been reported rarely in the literature. This case report presents a 48-year-old female with lymph node metastasis of carcinoma of the right mandibular posterior alveolar region concurrently with metastasis of PTC (with primary lesion clinically undetected) encountered during cervical lymph node examination.

## Introduction

In the adult population, at least 1% of individuals carry metastatic malignant neoplasms within their cervical lymph nodes. Notably, among patients with head and neck neoplasms, approximately 0.7% exhibit lymph nodes that house clinically occult but histologically malignant metastatic thyroid carcinomas [1]. The literature has documented approximately 49 cases of coexisting head and neck squamous cell carcinoma (HNSCC) and papillary thyroid carcinoma (PTC) [1]. PTC nodal metastasis is relatively common, with an occurrence rate of 1-10% in the population, as indicated by autopsy studies showing an incidence ranging from 6% to 35% [2]. Oral squamous cell carcinoma (OSCC) accounts for nearly 90% of malignant carcinomas arising from the oral mucosa region with a tendency to metastasize to cervical lymph nodes [3]. In patients with HNSCC, the co-incidental detection of secondary tumors like differentiated thyroid carcinoma within the cervical lymph nodes is rare [4]. This case report highlights a case of a female patient who presented with carcinoma of the right vestibular region with co-incidental nodal metastasis during lymph node examination following neck dissection.

## Case presentation

A 48-year-old female patient presented with pain and swelling in the right lower back tooth region for the past 10 days. One month back, the patient felt pain in the right lower back tooth region for which she underwent 45 extractions, after which she developed a painful growth in that region. She had no relevant medical or previous surgical history.

**Figure 1:**
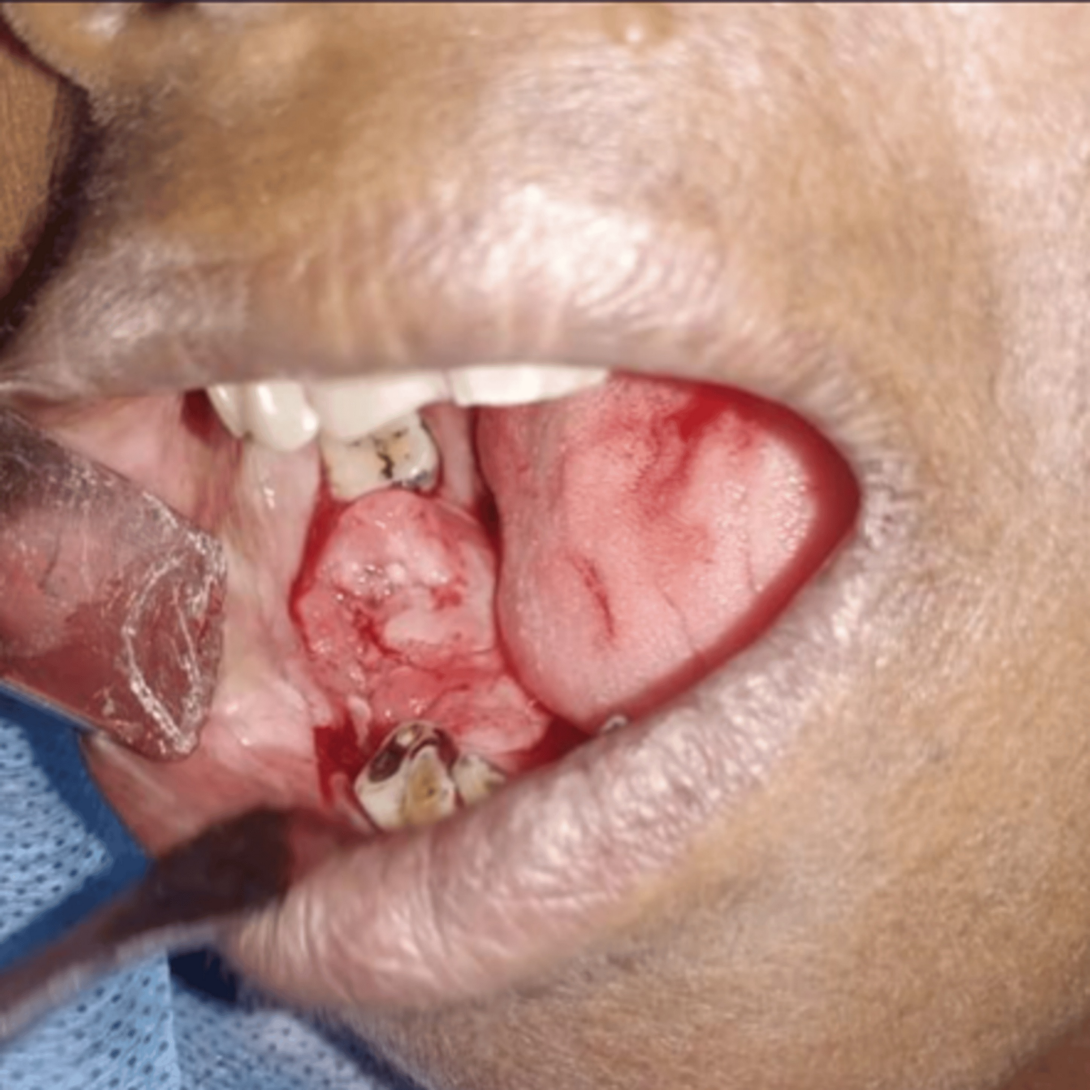
Proliferative growth in the right lower posterior alveolar mucosa of the 45, 46, and 47 regions.

**Figure 2:**
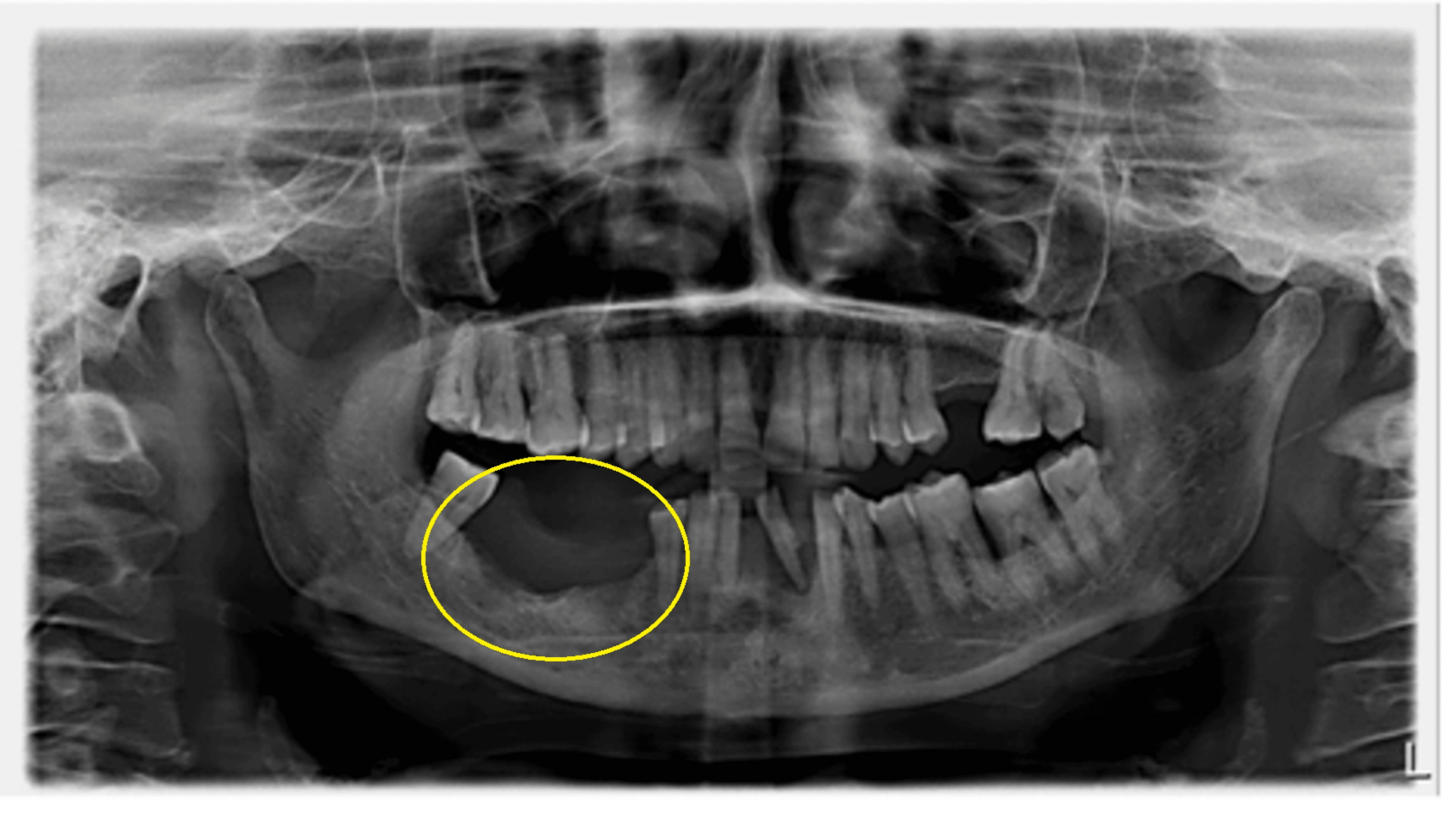
Orthopantomogram (OPG) showing radiolucency involving the right mandible edentulous area of 45, 46, and 47.

On extraoral examination, gross facial asymmetry was seen due to diffuse swelling in relation to the body of the mandible; on palpation, the swelling was hard in consistency and non-tender. A proliferative growth of size 3x2 cm was noticed in the alveolus of teeth 45, 46, and 47 with irregular surface, ulceration, and induration (Figure 1). An orthopantomogram (OPG) radiographic image showed radiolucency with a sclerotic border in the right lower mandible (Figure 2).

A CT examination revealed a heterogeneous lesion involving the vestibular mucosa near the right lower third molar region, extending and infiltrating the body of the right hemimandible and adjacent soft tissue. A provisional diagnosis of carcinoma of the posterior alveolar mandible was made. An incisional biopsy was performed and histopathologically was diagnosed as poorly differentiated OSCC (PDSCC).

**Figure 3:**
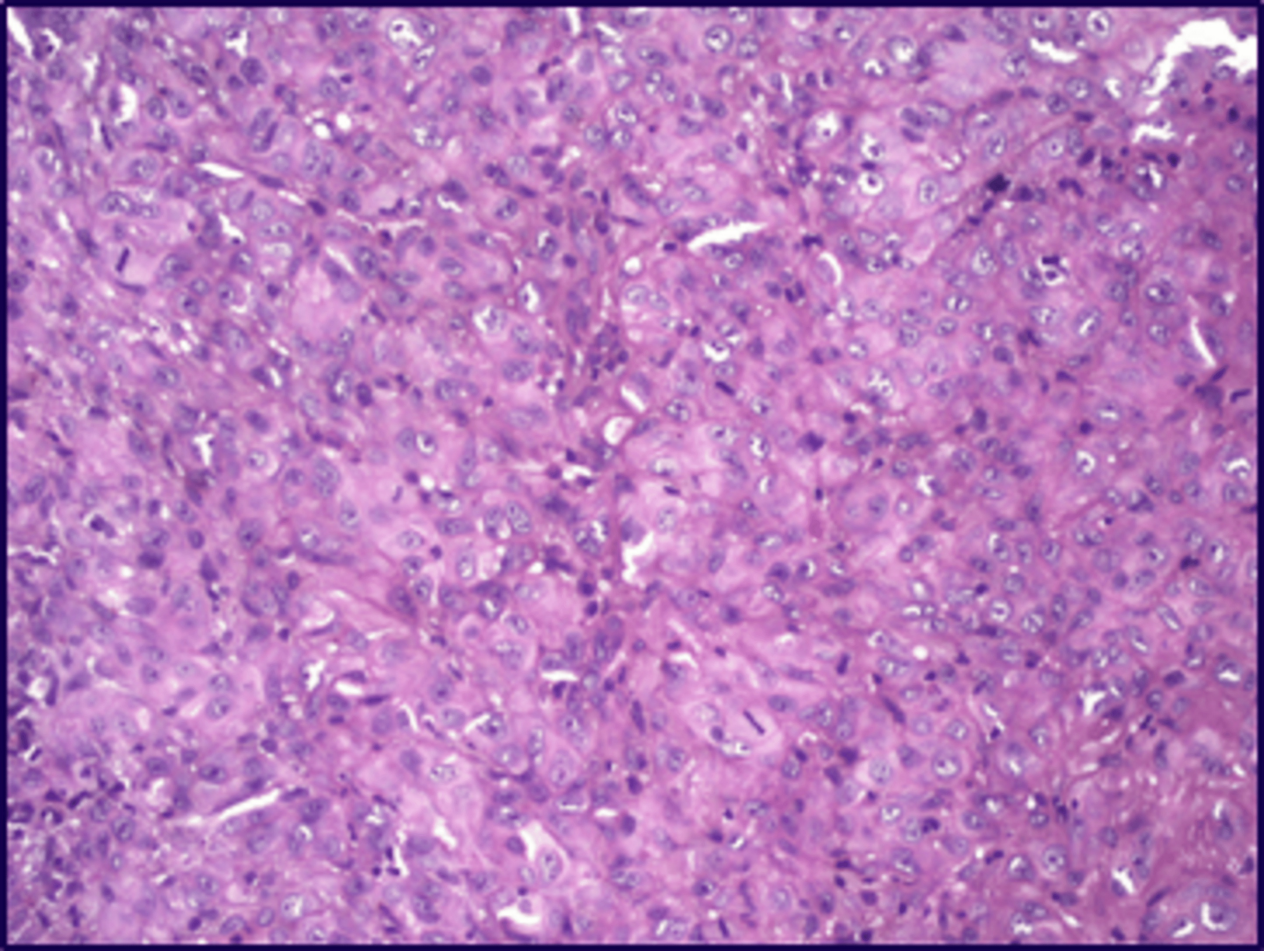
Histopathological image showing poorly differentiated oral squamous cell carcinoma.

The patient underwent a wide local excision with segmental mandibulectomy along with right radical neck dissection suspecting nodal metastasis. On histopathologic analysis, the sections of the soft tissue specimen revealed dysplastic surface stratified squamous epithelium with groups and loosely dispersed tumour cells invading most of the underlying fibro-cellular connective tissue with dispersed blood vessels and inflammatory cells (Figure 3). Sheets of tumour cells are seen invading the deeper areas involving muscles and around the nerves (perineural invasion). Focal areas of necrosis/myxoid degeneration were also seen.

**Figure 4:**
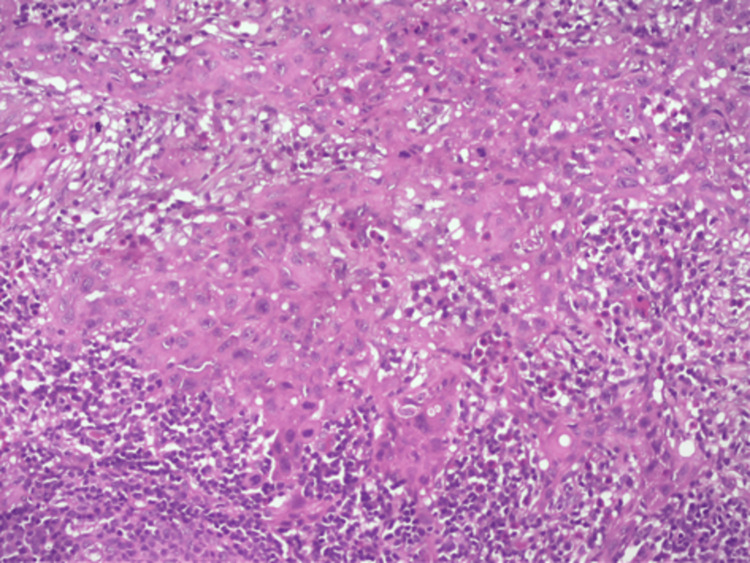
Histopathological image showing nodal invasion of tumour cells.

**Figure 5:**
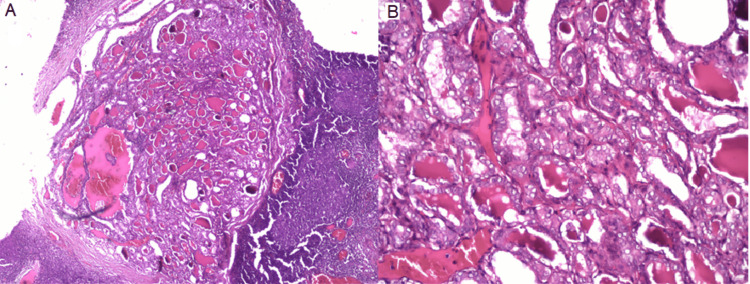
Metastatic papillary thyroid carcinoma in cervical lymph nodes (A: 10x, B: 20x).

Histopathological examination revealed four metastatic positive nodes out of 21 harvested ipsilateral lymph nodes (Figure 4). One node in levels III, IV, and V showed loss of the capsule with proliferating cells in a papillary fashion, displaying a nucleus with marginal chromatin and a bland centre, resembling Orphan Annie-eye clear nuclei. In some regions, cells were seen lining the central area showing an eosinophilic colloidal substance overall resembling thyroid tissue (Figure 5).

The other cortical and paracortical regions of the lymph node show lymphoid follicles, large blood vessels, adipose tissues, areas of haemorrhage, and extensive infiltration of metastatic thyroid carcinoma cells, which display classic papillary architecture (follicular type) in lymph nodes of III, IV, and V levels. Following histopathological reporting, the patient was referred for a CT scan investigation and the primary thyroid tumour was surgically excised. 

## Discussion

Cervical nodal metastasis accompanies nearly 50% of patients diagnosed with squamous cell carcinoma in the head and neck region [5]. The incidental finding of metastatic thyroid carcinoma in cervical lymph nodes during neck dissection for HNSCC has a reported prevalence rate of 0.3% to 1.6% according to the literature [6]. An incidental identification of metastatic PTC, during neck dissection, is considered rare, with an estimated incidence of less than 0.5% according to studies. Distinct histological changes in cervical lymph nodes raise concern for malignant metastases from primary tumours like those of the oesophagus, breast, stomach, lung, prostate, head-neck, and thyroid [7].

Cervical metastases are reported in approximately 50% of small PTC tumours and 75% of larger ones. In the case of our patient, the incidental finding of PTC metastasis in a cervical lymph node during the evaluation of OSCC raises important considerations. The lymph nodes most frequently affected by metastatic thyroid carcinoma are those at levels II, III, and IV, similar to our case [8]. Therefore, comprehensive sampling and meticulous examination of all specimens are imperative for an accurate diagnosis. The identification of PTC in this context underscores the necessity of a multidisciplinary approach involving otolaryngologists, oncologists, pathologists and endocrinologists to guide appropriate treatment strategies and optimize prognostic outcomes [9].

To the best of our knowledge, approximately 49 cases of coexistent HNSCC and PTC have been described in the literature [7]. Butler et al. reported that up to 3% of patients with head and neck cancer may have clinically undetected thyroid cancer [10]. Périé et al. recommended computed tomography and/or ultrasound examination of the neck and thyroid to look for a primary thyroid tumour during follow-up [11]. Despite variations in histological characteristics of thyroid tissue involvement, the prognosis remains favourable, with a reported 10-year survival rate exceeding 90%, and metastasis does not significantly impact overall outcomes, although older patients may face elevated risks, as suggested by existing literature [4]. Gerard-Marchant et al. describe benign thyroid inclusions as small clusters of histologically normal thyroid follicles within lateral cervical lymph nodes. While typically benign, they have a 0.03% incidence of potentially undergoing malignant transformation [12]. Accidentally discovered metastatic thyroid lesions in lymph nodes are less significant compared to more aggressive primary squamous cell tumours. Therefore, greater focus should be directed towards managing the primary tumour [13]. In our follow-up of the case, we are encouraged to report no evidence of recurrence of both OSCC and PTC in the past six months, indicating a favourable prognosis despite the initial discovery of metastases of thyroid carcinoma during neck dissection for HNSCC. Previous reports have similarly highlighted promising outcomes in such cases. Close surveillance and long-term follow-up are imperative to monitor for disease recurrence or metastasis from the primary tumour [14].

## Conclusions

This unique case underscores the importance of thorough evaluation and multidisciplinary collaboration in managing patients with concurrent malignancies. Clinicians should maintain a high index of suspicion for synchronous tumours, especially when dealing with cervical lymph node metastases. The coexistence of OSCC and incidental PTC in cervical lymph nodes necessitates vigilant diagnostic approaches and tailored treatment strategies.
